# Supplementary material for: Density-dependence tips the change of plant–plant interactions under environmental stress
Source: Nat Commun. 2020 May 21;11:2532. doi: 10.1038/s41467-020-16286-6 (PMC7242385; doi:10.1038/s41467-020-16286-6)
Supplement: Supplementary file 1 — Supplementary Information [file 41467_2020_16286_MOESM1_ESM.pdf]

Supplementary Information for

Density–dependence tips the change of plant–plant interactions under environmental stress

Ruichang Zhang and Katja Tielbörger

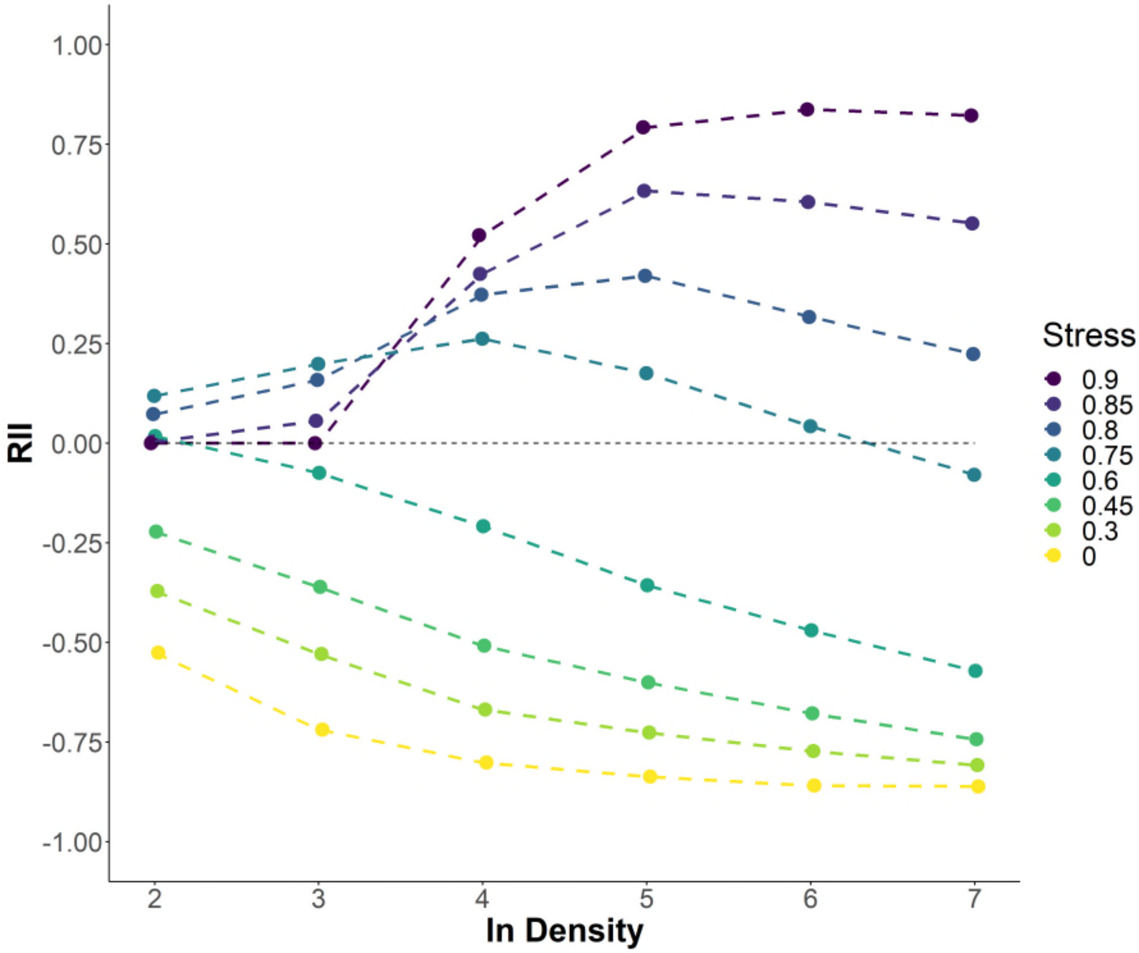

Supplementary Figure 1 Relationships between initial density (in ln scale) and Relative Interaction Index (RII) in simulated populations growing along a stress gradient. Source data are provided as a Source Data file.

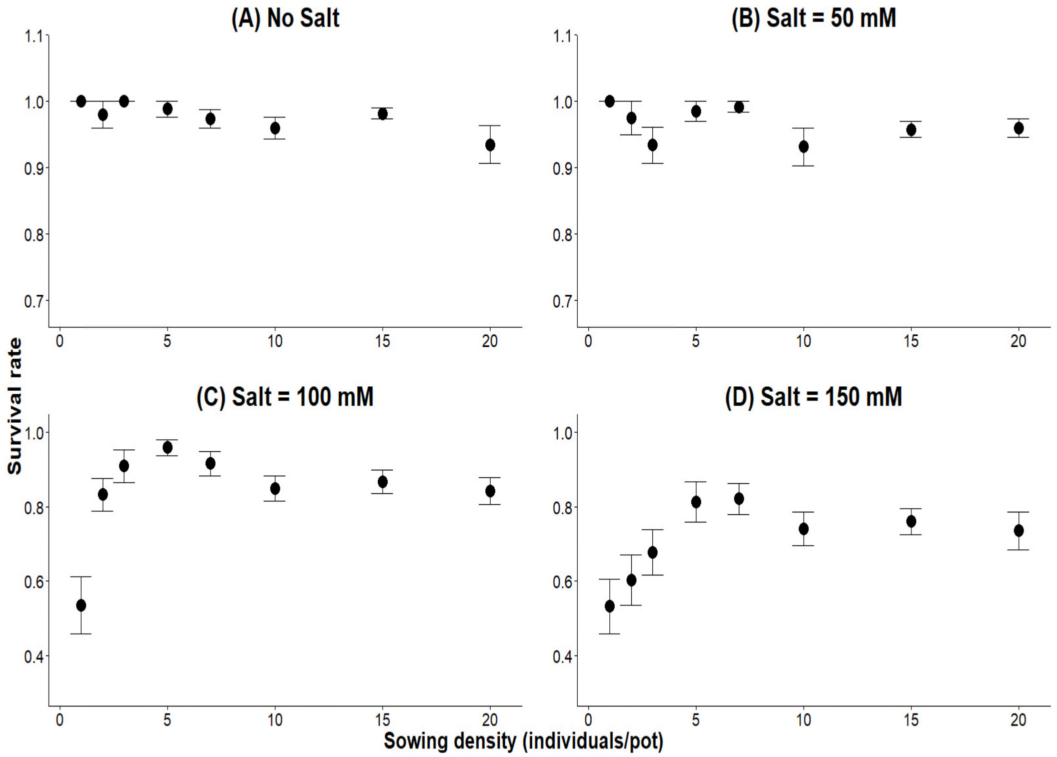

Supplementary Figure 2 Relationships between survival rate and initial density for *Arabidopsis thaliana* grown along a salinity gradient in a greenhouse experiment. Panel A – D for salt = 0, 50, 100 and 150 mM, respectively. Data are presented as mean values +/- SEM. For the density gradient from 2 to 20 plants per pot, n_{salt_0} = 25, 29, 17, 16, 15, 15 and 16 independent pots, respectively; n_{salt_50} = 20, 25, 13, 17, 16, 13, 16; n_{salt_100} = 30, 26, 15, 12, 14, 14, 13; n_{salt_150} = 29, 30, 15, 16, 17, 12, 11. Source data are provided as a Source Data file.

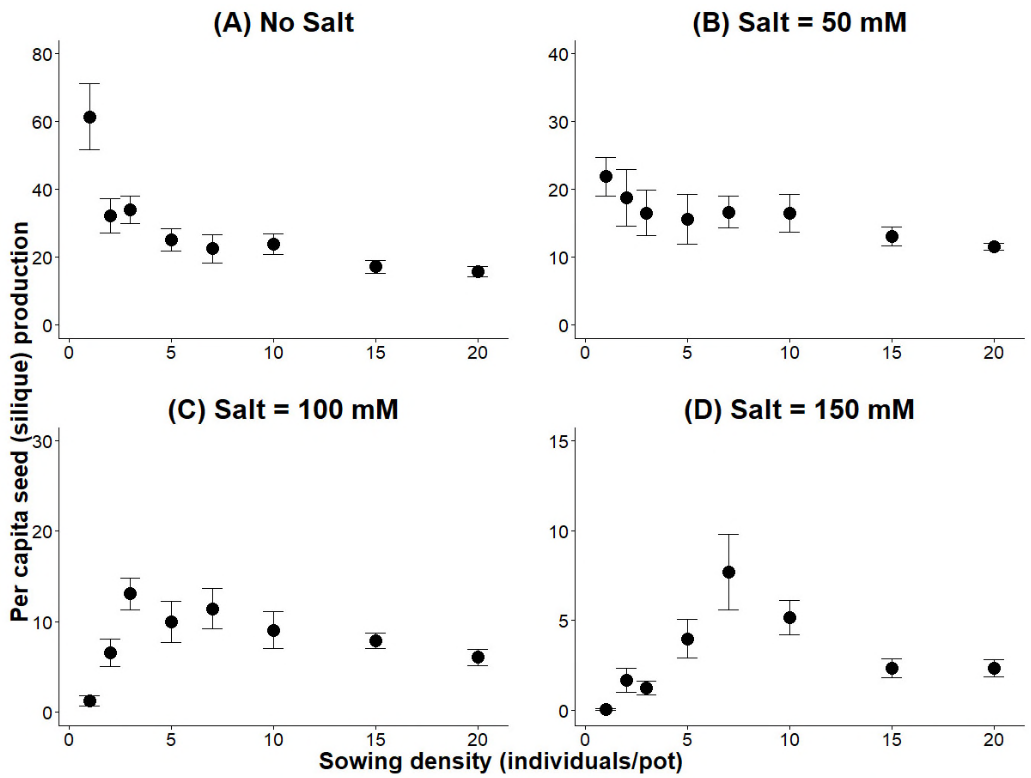

Supplementary Figure 3 Relationships between seed production and initial density for *Arabidopsis thaliana* grown along a salinity gradient in a greenhouse experiment. Panel A – D for salt = 0, 50, 100 and 150 mM, respectively. Data are presented as mean values +/- SEM. For the density gradient from 2 to 20 plants per pot, n_{salt_0} = 25, 29, 17, 16, 15, 15 and 16 independent pots, respectively; n_{salt_50} = 20, 25, 13, 17, 16, 13, 16; n_{salt_100} = 30, 26, 15, 12, 14, 14, 13; n_{salt_150} = 29, 30, 15, 16, 17, 12, 11. Source data are provided as a Source Data file.

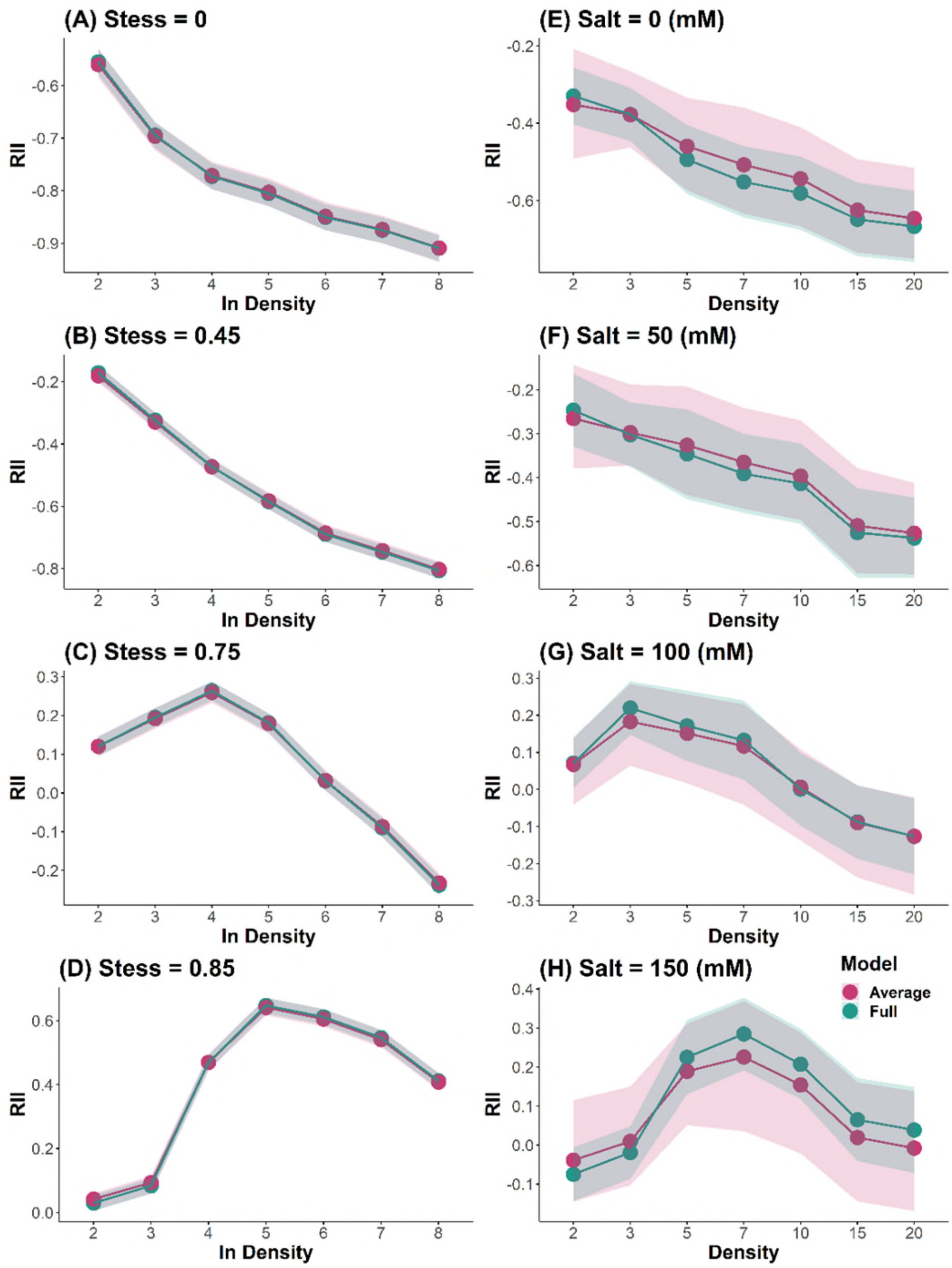

Supplementary Figure 4 Bayesian estimates of the Relative Interaction Index (RII; data are presented as mean and associated 95% credible set/confidence interval) of the averaged model and the full model. Panel A – D show the model simulations and n = 5 independent samples for the density gradient from 2 to 8 at each stress level (stress = 0, 0.45, 0.75 and 0.85 for Panel A – D, respectively); Panel E – H show the experiment and for the density gradient from 2 to 20 plants per pot, n_{salt_0} = 25, 29, 17, 16, 15, 15 and 16 independent pots, respectively; n_{salt_50} = 20, 25, 13, 17, 16, 13, 16; n_{salt_100} = 30, 26, 15, 12, 14, 14; n_{salt_150} = 29, 30, 15, 16, 17, 12, 11 (salt = 0, 50, 100 and 150 for Panel E – H, respectively). Magenta and green colors represent estimates based on the averaged and full model, respectively. Source data are provided as a Source Data file.

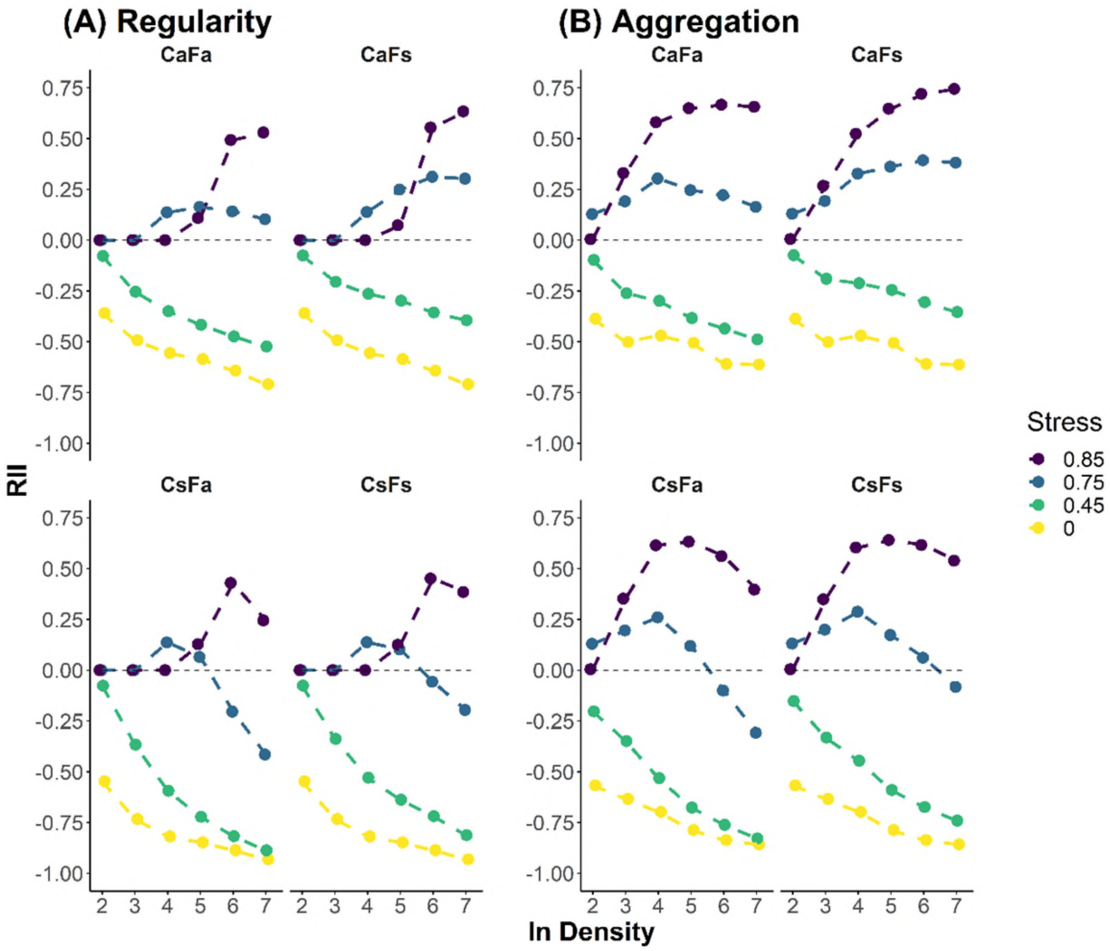

Supplementary Figure 5 Effects of different spatial distribution and interaction modes on RII–density relationships for simulated populations growing along a stress gradient. Ca = asymmetric competition; Cs = symmetric competition; Fa = asymmetric facilitation; Fs = symmetric facilitation. Panel A and B show the regularity and aggregation spatial pattern, respectively. Source data are provided as a Source Data file.

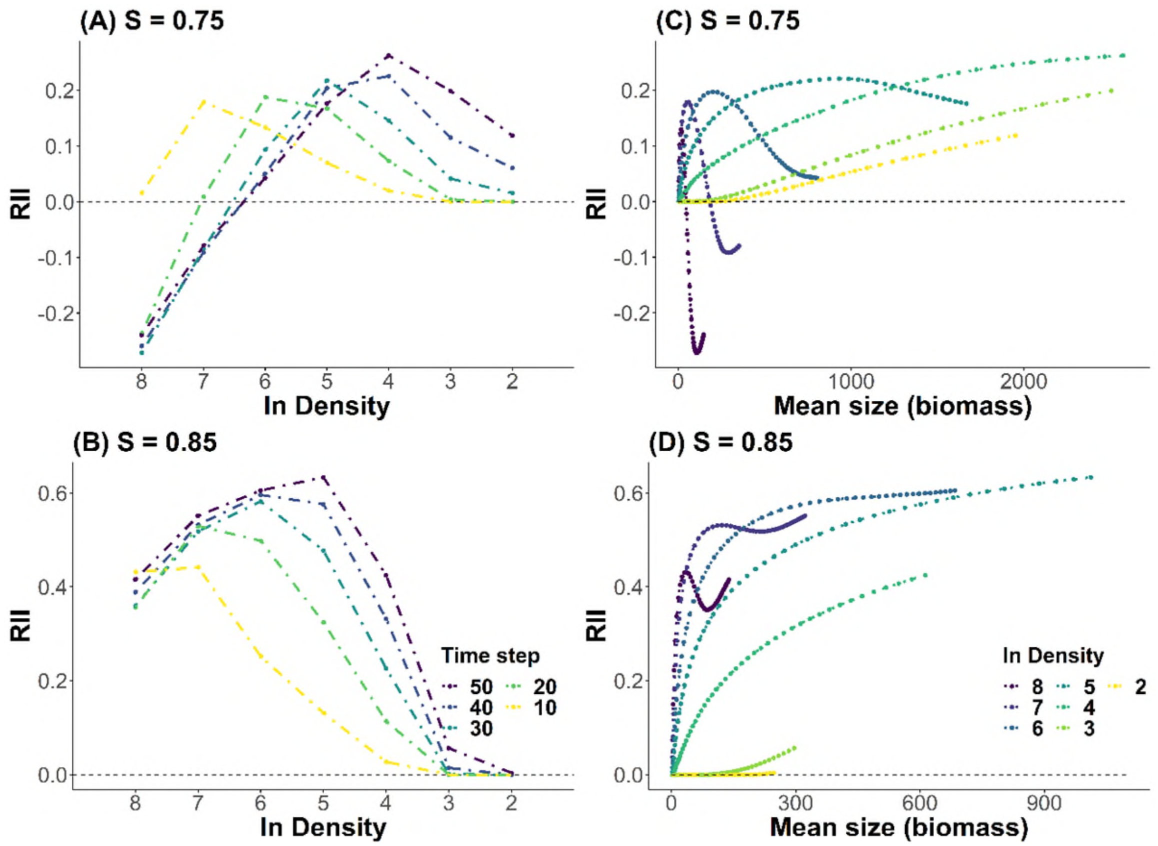

Supplementary Figure 6 Temporal dynamics of facilitation in the model simulations. Panel A and B show the RII–density relationship at every ten time steps under high (S = 0.75) and extreme stress (S = 0.85), respectively; Panel C and D show changes of RII during the development of plants at different initial densities under high (S = 0.75) and extreme stress (S = 0.85), respectively. Source data are provided as a Source Data file.

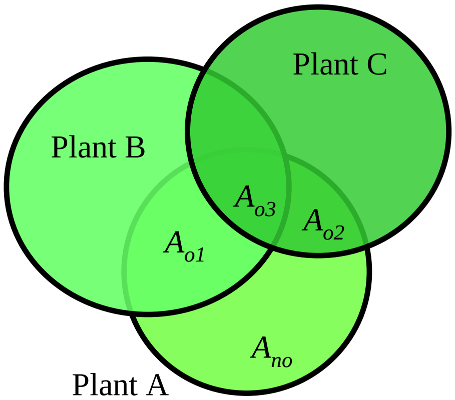

Supplementary Figure 7 Schematic illustration of interacting individuals (plant A, B and C with sizes m_1, m_2 and m_3, respectively) in a zone-of-influence (ZOI) model. Plant A is the target individual and its ZOI can be separated into four parts: A_{no} (non-overlapping area), A_{o1} (A and B), A_{o2} (A and C), A_{o3} (A, B and C).

Supplementary Table 1 Bayesian estimates of parameters and credible sets for the full model (density, stress and their interactions) predicting plant–plant interactions from density and stress.

Simulations					Experiment				
**Parameters**	**Estimate**	**Credible sets**	**Rhat**	**ESS**	**Parameters**	**Estimate**	**Credible sets**	**Rhat**	**ESS**
β_{0}(Intercept)	-0.55	(-0.58, -0.53)	1	10951	β0'(Intercept)	-0.33	(-0.42, -0.24)	1	14945
β_{1}(D3)	-0.14	(-0.18, -0.1)	1	14567	β1'(D3)	-0.05	(-0.17, 0.07)	1	18695
β_{2}(D4)	-0.22	(-0.26, -0.18)	1	15919	β2'(D5)	-0.16	(-0.3, -0.03)	1	19857
β_{3}(D5)	-0.25	(-0.29, -0.21)	1	15684	β3'(D7)	-0.22	(-0.36, -0.08)	1	20490
β_{4}(D6)	-0.3	(-0.34, -0.25)	1	15236	β4'(D10)	-0.25	(-0.39, -0.11)	1	20935
β_{5}(D7)	-0.32	(-0.36, -0.28)	1	15648	β5'(D15)	-0.32	(-0.46, -0.18)	1	20912
β_{6}(D8)	-0.35	(-0.4, -0.31)	1	15264	β6'(D20)	-0.34	(-0.48, -0.2)	1	19612
β_{7}(S0.45)	0.38	(0.34, 0.43)	1	13134	β7'(S50)	0.08	(-0.05, 0.21)	1	17059
β_{8}(S0.75)	0.68	(0.63, 0.72)	1	13839	β8'(S100)	0.4	(0.28, 0.52)	1	17262
β_{9}(S0.85)	0.58	(0.54, 0.63)	1	13644	β9'(S150)	0.25	(0.13, 0.38)	1	16600
β_{10}(D3S0.45)	-0.01	(-0.07, 0.05)	1	18199	β10'(D3S50)	-0.01	(-0.19, 0.17)	1	20970
β_{11}(D4S0.45)	-0.08	(-0.14, -0.03)	1	18206	β11'(D5S50)	0.06	(-0.14, 0.28)	1	23063
β_{12}(D5S0.45)	-0.16	(-0.22, -0.11)	1	19022	β12'(D7S50)	0.08	(-0.12, 0.28)	1	23578
β_{13}(D6S0.45)	-0.22	(-0.28, -0.16)	1	17955	β13'(D10S50)	0.08	(-0.12, 0.29)	1	23256
β_{14}(D7S0.45)	-0.26	(-0.31, -0.2)	1	18377	β14'(D15S50)	0.04	(-0.17, 0.25)	1	23921
β_{15}(D8S0.45)	-0.28	(-0.34, -0.22)	1	18009	β15'(D20S50)	0.05	(-0.16, 0.25)	1	21803
β_{16}(D3S0.75)	0.21	(0.16, 0.27)	1	18043	β16'(D3S100)	0.2	(0.03, 0.37)	1	21703
β_{17}(D4S0.75)	0.36	(0.3, 0.42)	1	18711	β17'(D5S100)	0.26	(0.07, 0.46)	1	23538
β_{18}(D5S0.75)	0.31	(0.25, 0.37)	1	19011	β18'(D7S100)	0.28	(0.08, 0.49)	1	25184
β_{19}(D6S0.75)	0.21	(0.15, 0.27)	1	18916	β19'(D10S100)	0.18	(-0.02, 0.38)	1	24584
β_{20}(D7S0.75)	0.11	(0.05, 0.17)	1	18873	β20'(D15S100)	0.16	(-0.04, 0.36)	1	24392
β_{21}(D8S0.75)	0	(-0.06, 0.06)	1	18697	β21'(D20S100)	0.14	(-0.06, 0.34)	1	24587
β_{22}(D3S0.85)	0.19	(0.13, 0.25)	1	18443	β22'(D3S150)	0.1	(-0.06, 0.27)	1	20799
β_{23}(D4S0.85)	0.66	(0.6, 0.72)	1	18784	β23'(D5S150)	0.46	(0.27, 0.66)	1	22234
β_{24}(D5S0.85)	0.87	(0.81, 0.93)	1	19422	β24'(D7S150)	0.58	(0.38, 0.78)	1	24667
β_{25}(D6S0.85)	0.88	(0.82, 0.94)	1	18849	β25'(D10S150)	0.53	(0.34, 0.73)	1	24147
β_{26}(D7S0.85)	0.84	(0.78, 0.9)	1	19260	β26'(D15S150)	0.46	(0.25, 0.67)	1	24466
β_{27}(D8S0.85)	0.74	(0.68, 0.79)	1	18111	β27'(D20S150)	0.45	(0.24, 0.66)	1	23908

Supplementary Table 2 Abbreviations for variables in the individual-based model (IBM).

**Symbol**	**Definition**	**Unit**
A	area of the circular zone-of-influence (ZOI)	cm^2
A_f	effective area with respect to facilitation	cm^2
A_h	cross-sectional area of heartwood at the ground base	cm^2
A_{no}	non-overlapping area of the target individual	cm^2
A_{oi}	the *i*th overlapping area	cm^2
B_0	constant for a given taxon, $B_l = B_0 m^{3/4}$	$W/g^{3/4}$
B_l	total incoming rate of light energy	W
B_c	average metabolic rate of a living cell	W
C	normalization constant, $B_l = CA$	W/cm^2
C_0	normalization constant, $A = C_0 m^{3/4}$	$cm^2/ g^{3/4}$
E_m	metabolic rate for building one unit of new tissue (per unit time)	$Wg^{-1}day^{-1}$
H	height, i.e. the length of the plant stem	cm
I_c	index for competition (resource) in the IBM	dimensionless
I_f	index for facilitation (stress) in the IBM	dimensionless
M	theoretical maximum body size of plants	g
N_c	number of living cells of plants	dimensionless
r	rate of building biomass per unit area and time, $r = a/C_0$	mg cm^{-2} day^{-1}
S	intensity of stress	dimensionless
V_h	volume of heartwood of woody plants	cm^3
a	constant for a given taxon, $a = B_0/E_m$,	$g^{1/4}$/day
b	constant, $b = B_c/(m_c E_m)$	day^{-1}
b'	constant, $b' = b - b_1$	dimensionless
b_1	normalization constant, $m_{dead} = b_1 m$	dimensionless
d_h	density of heartwood	g/cm^3
k	*k* plants cover in a certain overlapping area	dimensionless
m	total biomass of plants	g
m_c	average mass of a living cell	g
m_{dead}	biomass of dead tissue: mainly heartwood in the stem	g
m_{live}	biomass of live tissues	g
n	number of overlapping areas for the target individual	dimensionless
p	degree of size-symmetry of competition, ranges from 0 to ∞	dimensionless
q	degree of size-symmetry of facilitation, ranges from 0 to ∞	dimensionless

Supplementary Table 3 Variables used in the model simulations.

Variable	Definition (unit)	Values
S	Stress level: proportional reduction in incoming energy per unit of ZOI compared to a non-stress scenario (dimensionless proportion)	0, 0.3, 0.45, 0.6, 0.75, 0.8, 0.85, 0.9
Density	Number of individuals in the entire grid (number)	2 to 7 on logarithm scale (8 – 1089 individuals)
p	Mode of competition (continuous variable, dimensionless)	1 (symmetric) or 2(asymmetric)
q	Mode of facilitation (continuous variable, dimensionless)	1 (symmetric) or 2(asymmetric)
Spatial pattern	Initial distribution of individuals (category)	random, regular and aggregated
m	Plant biomass (mg)	initial biomass 2 ± 0.2
M	Theoretical maximum mass (mg)	$2\times10^6 \pm 2\times10^5$
r	Intrinsic growth rate (mg cm^{-2} day^{-1})	3 ± 0. 3
A	Area of Zone Of Influence (cm^2)	$m^{3/4}$

Supplementary Methods

The Supplementary Method (below) is the detailed description of the mathematical models and it includes: 1) the derivation of the growth equation describing the potential growth rate of single plants; 2) the individual-based model (IBM) by incorporating competition and facilitation explicitly; 3) an illustration for our approach to including both facilitative effect and response in the model; 4) an example for the specific calculation of the model; and 5) details about the model simulations.

The growth equation is based on a commonly adopted general energy conservation Equation (1-3):

$$\boldsymbol{B_l = B_c N_c + E_m \frac{dm}{dt}} \quad \textbf{(1)}$$

where B_l denotes the total incoming rate of light energy flow (gross photosynthetic rate), B_c is the average metabolic rate of a living cell, N_c is the number of living cells and E_m represents the energy required for building one unit of new biomass per unit of time (differences among cells and tissues are ignored, see also Supplementary Table 2 for abbreviations). This equation reflects the idea that the total metabolic energy can be divided into two parts: one fraction is used to build and store new biomass (i.e. growth), and the remaining fraction is used to maintain existing tissues (2).

Non-woody and woody plants differ in the allocation of energy to these two types of processes, as the latter consist of both live and dead tissues. For example, foliage, fine roots and sapwood are usually live tissues, whereas the heartwood in the woody stem is dead and does not require metabolic energy for maintenance (1, 4). Therefore, the growth rate of total biomass (dm/dt) is the sum of two terms: one which denotes the rate of transition of live to dead biomass (dm_{dead}/dt), and a second one which denotes the net increase of biomass of live tissues (dm_{live}/dt):

$$\boldsymbol{\frac{dm}{dt} = \frac{dm_{live}}{dt} + \frac{dm_{dead}}{dt}} \quad \textbf{(2)}$$

where $m_{live} = m_c N_c$. The allometric relationship between the entire metabolic rate and body size is usually modelled as: $B_l = B_0 m^{3/4}$, where B_0 is constant for a given taxon and the 3/4 exponent is derived and well supported by many empirical studies (5-9).

Then, Equation (1) can be rewritten as:

$$\frac{dm}{dt} = \frac{B_0}{E_m} m^{3/4} - \frac{B_c}{m_c E_m}(m - m_{dead}) \quad \textbf{(3)}$$

Considering that we used an annual species in our greenhouse experiment (*Arabidopsis Thaliana*), biomass of dead tissues m_{dead} is *0* and the growth equation can be written as:

$$\frac{dm}{dt} = am^{3/4} - bm \quad \textbf{(4)}$$

where $a = B_0/E_m$, $b = B_c/(m_c E_m)$.

Although we focus on non-woody plants in our experiment, the growth equation could actually be easily expanded and applied to woody plants, too. For example, dead tissues (mostly heartwood) in woody plants are usually modeled as a cone (4, 10) and its volume can be approximately calculated as $V_h = 1/3 \times A_h H$, where A_h denotes the cross-sectional area of heartwood at the ground base and H is the length of the stem. Because the radius of the stem $r \propto m^{3/8}$ and $H \propto m^{1/4}$ (6, 11), we obtain the following scaling relationship: $V_h \propto 1/3 \times m^{(3/8)\times 2}\, m^{1/4} \propto m$. We assume that the density of the heartwood d_h is a species-specific constant, therefore, $m_{dead} = d_h V_h \propto m = b_1 m$ (b_1 is a normalization constant). Then the growth equation for woody plants can also be expressed as:

$$\frac{dm}{dt} = am^{3/4} - b'\, m \quad \textbf{(5)}$$

where $b' = b - b_1$. The structure of Equation (4) and (5) is similar to the general growth equation for animals (2), although the details of the parameters are different.

In the equations, the term representing maintenance (bm or $b'm$) always increases faster than the term of total incoming energy ($am^{3/4}$) taking into account that growth is not unlimited. Thus, all metabolic energy will eventually be allocated to sustain live issues when plants reach their maximum body size (M). Taking $dm/dt = 0$ gives $M = (a/b)^4$ or $M = (a/b')^4$. Thus, both Equation (4) and (5) can be rewritten as:

$$\frac{dm}{dt} = am^{\frac{3}{4}}\left[1 - \left(\frac{m}{M}\right)^{\frac{1}{4}}\right] \quad \textbf{(6)}$$

Consistent with empirical evidence (12), Equation (6) describes a sigmoidal growth of biomass. In stressful environments, we assume that the incoming energy ($am^{3/4}$) will be strongly decreased compared to benign environments because photosynthesis is the most fundamental physiological process in green plants and may be limited by almost all stress

factors (13). The growth equation in stressful environments can therefore be expressed as:

$$\frac{dm}{dt} = am^{\frac{3}{4}}\left[1 - S - \left(\frac{m}{M}\right)^{\frac{1}{4}}\right] \tag{7}$$

where S indicates the intensity of stress (the fraction of reduction in incoming energy caused by stress). For instance, $S = 0.3$ indicates that a plant in a stressful environments obtains 30% less energy than in benign conditions. S ranges from 0 (no stress) to 1 (extreme stress, plants cannot obtain any resource at all) but plants may stop growing when $1 - S = (m/M)^{0.25}$, i.e., the available energy can only be used to sustain live issues.

In order to investigate plant-plant interactions, the above growth equation was then combined with the zone-of-influence (ZOI) method. In a spatially-explicit ZOI model, each individual covers a circular zone in a two-dimensional space that can be understood as the physical zone where plants obtain resources and thus decrease resource availability for potential neighbors (14, 15). The area of this zone (A) reflects the maximum amount of energy and/or resource available for plants. Therefore, the total incoming rate of energy (B_l) will scale as A: $B_l \propto A = CA$, where C is a constant and denotes the energy that a plant can obtain per unit area and time. Because B_l is allometrically related to plant biomass ($B_l = B_0 m^{3/4}$), we can derive the relationship between A and plant biomass as follows: $A = C_0 m^{3/4}$, where $C_0 = B_0/C$. Therefore, in the ZOI model, Equation (7) can be re-written as:

$$\frac{dm}{dt} = rA\left[1 - S - \left(\frac{m}{M}\right)^{\frac{1}{4}}\right] \tag{8}$$

where $r = C/E_m$, is the rate of biomass increase per unit area and time, i.e. the intrinsic growth rate (in units of mass·area^{-1}·time^{-1}). In the ZOI model, neighboring plants interact with each other in areas where their ZOIs overlap. Including plant-plant interactions as two dimensionless indices (I_c for competition and I_f for facilitation) into Equation (8) leads to:

$$\frac{dm}{dt} = rA\left[(1 - SI_f)I_c - \left(\frac{m}{M}\right)^{\frac{1}{4}}\right] \tag{9}$$

This equation is the main one underlying our model (see below, details of model).

In Equation (9), I_c represents the proportion of available resources when an individual competes with neighbors:

$$\boldsymbol{I_c = \frac{A_c}{A} = \frac{A_{no} + \sum_{i=1}^{n} \frac{m^p}{\sum_{j=1}^{k} m_j^p} A_{oi}}{A}} \qquad \textbf{(10)}$$

where A_c is the effective area for resource acquisition and is calculated as the sum of non-overlapping area and the fraction of resources they can achieve in overlapping zones. A_{no} is the non-overlapping area of the target individual while n refers to the number of overlapping zones. A_{oi} is the area of ith overlapping zone and k denotes that the ith overlapping area is occupied by k plants. The division of A_{oi} is determined by the parameter p, which reflects the degree of size-symmetry of competition (16). It ranges from 0 (completely symmetric, all plants share the resource equally regardless of their sizes) to ∞ (completely asymmetric, the larger individual obtains all resource). Specifically, p=1 represents size-symmetric competition, i.e., the overlapping area (contested resource) is divided proportionally to the size of overlapping individuals and associated ZOIs.

In harsh conditions, the area of ZOI is also related to the stress that limits the plants, and in overlapping ZOIs, neighbours can potentially alleviate stress. Similarly, I_f represents the proportion of realized stress for plants when neighbors are present:

$$\boldsymbol{I_f = 1 - \frac{A_f}{A} = 1 - \frac{\sum_{i=1}^{n} \left(1 - \frac{m^q}{\sum_{j=1}^{k} m_j^q}\right) A_{oi}}{A}} \qquad \textbf{(11)}$$

where A_f indicates the effective facilitative area of neighbors (stress that is ameliorated by neighbors). Accordingly, A_f/A can be understood as the fraction of stress relieved by benefactors. Likewise, A_f is calculated as the sum of the effective facilitative area in each overlapping ZOI, which is determined by the parameter q. This parameter reflects the symmetry of facilitation (17) and it also ranges from 0 (completely symmetric, all plants contribute equally to stress amelioration irrespective of their size) to ∞ (completely asymmetric, only the larger plants suffer from stress and contribute to habitat amelioration). Specifically, p = 1 represents size-symmetric facilitation, i.e., plants are stressed proportionally to their size (18). An example for calculating I_c and I_f is provided below (see section 4).

As a pairwise interaction, facilitation depends on two aspects: the stress amelioration by benefactors (facilitative effect) and the sensitivity of beneficiaries to this stress amelioration (facilitative response). In our model, A_f indicates the alleviated stress by neighbors and is determined by the size of the overlapping areas and how they are divided (i.e., the mode of facilitation). In harsh conditions, stress is homogeneous across space, i.e., the proportional reduction in incoming resource per unit area is identical. The size of ZOI (*A*) actually reflects the 'quantity of stress' experienced by the individual and is thus key to determining sensitivity to stress mitigation. Specifically, the larger *A*, the more benefit is necessary for improving severe environmental conditions. Therefore, even if two conspecific individuals receive the same benefit, the smaller one will experience stronger facilitation because the benefit is relatively greater for its size (see Fig.5 in main text).

To reflect the influence of facilitation on plant performance, including facilitative effect and response should be equally important. However, all of the few previous models used the term $S/(1+ A_f)$ to model how stress and facilitation affect plant growth (18-20). For a given stress level, this term is only determined by facilitative effect which is proportional to the area covered by the benefactor (A_f), but the size of the beneficiary and thus its sensitivity is neglected. An example is illustrated in Fig.5 in the main text where plant A and B which differ in their ZOI (i.e. here: facilitative response) receive exactly the same absolute benefit, e.g. as shading from high radiation, from benefactor X ($A_{fa} = A_{fb}$). The plant with the small ZOI will not suffer from radiation at all while only 50% of the stress is alleviated for B. However, when we adopt the approach of the previous models, the influence of stress amelioration is the same for both beneficiaries ($S/(1+ A_{fa})= S/(1+ A_{fb})$). This is obviously not realistic and conflicts with empirical evidence. In fact, recent studies have already found that larger individuals often have lower sensitivity to environmental changes, thus reducing benefits of habitat amelioration by neighbors. In other words, smaller plants respond more positively to stress amelioration (21-23).

Another problem of all previous models is that by ignoring the size-dependence of facilitative response, the units on the two sides of the equations are not identical. More specifically, due to ignoring the sensitivity (determined by *A*, the unit of which is area), the unit of A_f (i.e., area) is not balanced in the term $S/(1+ A_f)$. Therefore, the unit on the

left side of the equation is mass·time^{-1} while the unit on the right side is mass·time^{-1}·area^{-1} (see Equation (3) in Lin et al. 2012) or even more complicated (mass·time^{-1}· (area^{-1} – mass$^{0.5}$·area^{-2}), see Equation (3) in Chu et al. 2008). More importantly, simulations based on these models showed that the interaction intensity-density curve simply moves upward or even to the left (results not shown). This apparently conflicts with basic reasoning but also with the pattern we observed in the parallel greenhouse experiment (i.e., a rightward shift, see main manuscript). This indicates that such models may be seriously flawed and that our approach is superior to previous ones.

Here, we used a novel term $SI_f = S(1- A_f/A)$ in the main equation to represent the influence of facilitation. Then the actual stress for plant A and B in our example (Fig.5 in the main text) is $S(1- A_{fa}/A_a)$ = 0 and $S(1- A_{fb}/A_b)$ = 0.5S, respectively. This term is obviously more realistic because it can integrate both facilitative effect of benefactors and sensitivity of beneficiary plants. Most importantly, including both effect and response yields identical units of both sides of the equation (mass·time^{-1} on both sides). This mathematically correct model also fits very well with the experimental results as well as with ecological reasoning.

In terms of plant A (Supplementary Fig.7), competition and facilitation co-occur in the overlapping areas A_{o1}, A_{o2} and A_{o3}. Then the resource (proportional to the effective area) it can achieve in each area is calculated as follows:

$$\frac{m_1^p}{\sum_{j=1}^{2} m_j^p}\boldsymbol{A_{o1}} = \frac{m_1^p}{m_1^p + m_2^p}\boldsymbol{A_{o1}} \text{ (for } A_{o1})$$

$$\frac{m_1^p}{\sum_{j=1}^{2} m_j^p}\boldsymbol{A_{o2}} = \frac{m_1^p}{m_1^p + m_3^p}\boldsymbol{A_{o2}} \text{ (for } A_{o2})$$

$$\frac{m_1^p}{\sum_{j=1}^{3} m_j^p}\boldsymbol{A_{o3}} = \frac{m_1^p}{m_1^p + m_2^p + m_3^p}\boldsymbol{A_{o3}} \text{ (for } A_{o3})$$

A_c is the sum of non-overlapping area (A_{no}) and of area it can cover in A_{o1}, A_{o2} and A_{o3}. Therefore, the index for competition (I_c) is calculated as:

$$\boldsymbol{I_c} = \frac{A_c}{A} = \frac{A_{no} + \frac{m_1^p}{m_1^p + m_2^p}A_{o1} + \frac{m_1^p}{m_1^p + m_3^p}A_{o2} + \frac{m_1^p}{m_1^p + m_2^p + m_3^p}A_{o3}}{A}$$

Similarly, the effective area covered by neighbors (the stress relieved by neighbors, i.e. benefits they can provide) in each area is calculated as follows:

$$\left(\mathbf{1}-\frac{m_1^q}{\sum_{j=1}^{2} m_j^q}\right)A_{o1}=\frac{m_2^q}{m_1^q+m_2^q}A_{o1}\ \text{(for } A_{o1}\text{)}$$

$$\left(\mathbf{1}-\frac{m_1^q}{\sum_{j=1}^{2} m_j^q}\right)A_{o2}=\frac{m_3^q}{m_1^q+m_3^q}A_{o2}\ \text{(for } A_{o2}\text{)}$$

$$\left(\mathbf{1}-\frac{m_1^q}{\sum_{j=1}^{3} m_j^q}\right)A_{o3}=\frac{m_2^q+m_3^q}{m_1^q+\ m_2^q+m_3^q}A_{o3}\ \text{(for } A_{o3}\text{)}$$

A_f is the sum of these effective areas and the index for facilitation (I_f) is calculated as:

$$I_f=\mathbf{1}-\frac{A_f}{A}=\mathbf{1}-\frac{\frac{m_2^q}{m_1^q+m_2^q}A_{o1}+\frac{m_3^q}{m_1^q+m_3^q}A_{o2}+\frac{m_2^q+m_3^q}{m_1^q+m_2^q+m_3^q}A_{o3}}{A}$$

The model was simulated in Netlogo (24). To simplify the calculation of effective areas, we used discrete patches (200 × 200 grid cells) to mimic a continuous two-dimensional space (15, 18). A torus was used to avoid edge effects (25). In the simulations, the process of plant growth was reflected as increasing areas of ZOIs over time. Growth followed the main equation, i.e. Equation (9), which means growth is determined by resource availability, competition intensity, abiotic stress intensity and facilitation intensity. Values of variables in Netlogo (Supplementary Table 3) were based on previous studies (18) and our preliminary simulations. To facilitate the interpretation of the results, conventional units were assigned to measures of biomass, growth rate and area (Supplementary Table 3).

In the simulations, we explored density (seven levels: from 2 to 8 on a ln scale); stress (eight levels: from 0 to 0.9), spatial pattern (random, regular and aggregated), and different modes of competition and facilitation. In the 'Results' of the main manuscript, we show only one scenario (the one most similar to the experiment), as the overall patterns were essentially the same. Specifically, we selected four stress levels (0, 0.45, 0.75, 0. 85), random spatial pattern, symmetric competition (p = 1) and symmetric facilitation (q = 1) for the main text because these settings were similar to our greenhouse experiment, e.g., with rosette plants and salt stress, competition and facilitation were more prone to be symmetric. The results of the other simulation scenarios can be found in Supplementary Fig.1 and 5 and they indicate no qualitative difference to the findings shown in the main text. For simulations focusing on RIIs and density, we collected data every ten time steps and present results after 50 steps because the interaction-density

relationships developed more strongly over time and stabilized after approx. 40 time steps. Moreover, the model has a defined growth rate for each individual. Thus, by recording changes of RIIs as plant sizes increased, we were able to look at whether plant size can affect the intensity of facilitation and how this was determined by the stress-gradient and by density simultaneously. To do so, we ran additional simulations to collect data of the mean biomass of populations under relatively high stress (S = 0.75 and 0.85) at each time step. Results including size can be seen in Supplementary Fig.6. They indicate that RIIs generally increased with size (biomass) while increased densities compensated for smaller size and reduced facilitation.

Values for intrinsic growth rate, initial plant biomass, and theoretical maximum plant biomass were randomly chosen from the intervals in Supplementary Table 3 to simulate natural variation among individuals. Individuals whose growth rate fell below a certain threshold (5% of $m^{3/4}$) were considered to be dead (17), i.e. they were unable to increase their ZOIs. To mimic our experiment where all individuals were harvested at the end of the experiment, we did not remove these dead plants until the end of each simulation. This also reflects better a natural situation, where e.g. shading can also be imposed by dead plants. It should be noted that the results were qualitatively similar to a scenario where dead plants were removed.

Supplementary References

1. Enquist BJ & Niklas K, Invariant scaling relations across tree-dominated communities. *Nature* **410**, 655-660 (2001).
2. West GB, Brown JH, & Enquist BJ, A general model for ontogenetic growth. *Nature* **413**, 628-631(2001).
3. Hou C, *et al.* Energy Uptake and Allocation During Ontogeny. *Science* **322**, 736-739 (2008).
4. Valentine HT & Mäkelä, A Bridging process-based and empirical approaches to modeling tree growth. *Tree Physiology* **25**, 769-779 (2005).
5. West GB, Brown JH, & Enquist BJ, The fourth dimension of life: fractal geometry and allometric scaling of organisms. *Science* **284**, 1677-1679 (1999).
6. West GB, Brown JH, & Enquist BJ, A general model for the structure and allometry

of plant vascular systems. *Nature* **400**, 664-667 (1999).
7. Enquist BJ, Brown J, & West G, Allometric scaling of plant energetics and population density. *Nature* **395**, 163-165 (1998).
8. Price CA, Enquist BJ, & Savage VM, A general model for allometric covariation in botanical form and function. *Proc. Natl. Acad. Sci. USA* **104**, 13204-13209 (2007).
9. Enquist BJ, West GB, & Brown JH, Extensions and evaluations of a general quantitative theory of forest structure and dynamics. *Proc. Natl. Acad. Sci. USA* **106**, 7046-7051 (2009).
10. Climent J, Chambel MR, Gil L, & Pardos JA, Vertical heartwood variation patterns and prediction of heartwood volume in Pinus canariensis Sm. *For. Ecol. Manage.* **174**, 203-211 (2003).
11. Savage VM, *et al.* Hydraulic trade-offs and space filling enable better predictions of vascular structure and function in plants. *Proc. Natl. Acad. Sci. USA* **107**, 22722-22727 (2010).
12. Weraduwage SM, *et al.* The relationship between leaf area growth and biomass accumulation in Arabidopsis thaliana. *FRONT PLANT SCI* doi: 10.3389/fpls.2015.00167 (2015).
13. Ashraf M & Harris PJC, Photosynthesis under stressful environments: An overview. *Photosynthetica* **51**, 163-190 (2013).
14. Stoll P & Bergius E, Pattern and process: competition causes regular spacing of individuals within plant populations. *J. Ecol.* **93**, 395-403 (2005).
15. Weiner J, Stoll P, Muller - Landau H, & Jasentuliyana A, The Effects of Density, Spatial Pattern, and Competitive Symmetry on Size Variation in Simulated Plant Populations. *AM NAT* **158**, 438-450 (2001).
16. Schwinning S & Weiner J, Mechanisms determining the degree of size asymmetry in competition among plants. *Oecologia* 113, 447-455 (1998).
17. Lin Y, Berger U, Grimm V, & Ji Q-R, Differences between symmetric and asymmetric facilitation matter: exploring the interplay between modes of positive and negative plant interactions. *J. Ecol.* **100**, 1482-1491 (2012).
18. Lin Y, Berger U, Yue M, & Grimm V, Asymmetric facilitation can reduce size

inequality in plant populations resulting in delayed density-dependent mortality. *Oikos* **125**, 1153-1161 (2016).

19. Chu C-J, *et al.* Balance between facilitation and resource competition determines biomass–density relationships in plant populations. *Ecol. Lett.* **11**, 1189-1197 (2008).
20. Zhang WP, *et al.* The interplay between above- and below-ground plant-plant interactions along an environmental gradient: insights from two-layer zone-of-influence models. *Oikos* **122**,1147-1156 (2013).
21. Schiffers K & Tielbörger K, Ontogenetic shifts in interactions among annual plants. *J. Ecol.* **94**, 336-341 (2006).
22. Sthultz CM, Gehring CA, & Whitham TG, Shifts from competition to facilitation between a foundation tree and a pioneer shrub across spatial and temporal scales in a semiarid woodland. *New Phytol.* **173**, 135-145 (2007).
23. le Roux PC, Shaw JD, & Chown SL, Ontogenetic shifts in plant interactions vary with environmental severity and affect population structure. *New Phytol.* **200**, 241-250 (2013).
24. Wilensky U (1999) Netlogo. http://ccl.northwestern.edu/netlogo/. Center for Connected Learning and Computer-Based Modeling, Northwestern University. Evanston, IL.
25. Grimm V & Railsback SF, *Individual-based modeling and ecology.* Princeton university press (2005).
